# Global population surpasses eight billion: Are we ready for the next billion?

**DOI:** 10.3934/publichealth.2023056

**Published:** 2023-10-25

**Authors:** Nityanand Jain, Islam Kourampi, Tungki Pratama Umar, Zahra Rose Almansoor, Ayush Anand, Mohammad Ebad Ur Rehman, Shivani Jain, Aigars Reinis

**Affiliations:** 1 Faculty of Medicine, Riga Stradinš University, 16 Dzirciema street, Riga, Latvia, LV-1007; 2 Department of Medicine, National and Kapodistrian University of Athens, Mikras Asias 75, Athens, Greece, 11527; 3 UCL Centre for Nanotechnology and Regenerative Medicine, Division of Surgery and Interventional Science, University College London, London, United Kingdom; 4 Faculty of Medicine, Universitas Sriwijaya, Palembang, Indonesia 30128; 5 Faculty of Biology, Medicine and Health, The University of Manchester, Oxford Rd, Manchester M13 9PL, United Kingdom; 6 BP Koirala Institute of Health Sciences, Buddha Road, Dharan 56700, Nepal; 7 Faculty of Medicine, Rawalpindi Medical University, Rawalpindi, Pakistan 46000; 8 Department of Oral and Maxillofacial Surgery, Genesis Institute of Dental Sciences & Research, Ferozepur-Moga Road, Ferozepur, Punjab, India 152002; 9 Department of Biology and Microbiology, Riga Stradinš University, 16 Dzirciema street, Riga, Latvia, LV-1007

**Keywords:** population, health policy, universal health coverage, food, water, noncommunicable diseases

## Abstract

In November 2022, the global population had officially crossed eight billion. It has long been recognized that socioeconomic or health-related problems in the community always accompany an uncontrolled population expansion. International calls have been made regarding lack of universal health coverage, an insufficient supply of healthcare providers, the burden of noncommunicable disease, population aging and the difficulty in obtaining safe drinking water and food. The present health policy paper discusses how to conquer these crowded world issues, including (1) promoting government and international organization participation in providing appropriate infrastructure, funding and distribution to assist people's health and well-being; (2) shifting health program towards a more preventive approach and (3) reducing inequalities, particularly for the marginalized, isolated and underrepresented population. These fundamental principles of health policy delivery as a response to an increasingly crowded world and its challenges are crucial for reducing the burden associated with excessive healthcare costs, decreased productivity and deteriorating environmental quality.

*“This year (2022) World Population Day falls during a milestone year, when we anticipate the birth of the Earth's eight billionth inhabitant* (on 15^th^ November 2022). *This is an occasion to celebrate our diversity, recognize our common humanity, and marvel at advancements in health....... At the same time, it is a reminder of our shared responsibility to care for our planet and a moment to reflect on where we still fall short of our commitments to one another”*

*UN Secretary-General H.E. Antonio Guterres, November 2022*

According to modelled estimates, November 15^th^, 2022, celebrated the birth of the eight billionth human being on the planet – marking an addition of a billion people within about eleven years [1]. The United Nations (UN) estimates that by the end of the century, the number of people living on Earth will have surpassed 10 billion with approximately 227,000 babies being brought to life daily [2]. Despite the positive demographic trends, the issues and challenges that have been called out and talked about at various forums, meetings and UN General Assemblies over the years still remain to be tackled. It's been more than 40 years since the signing of the Alma Ata Declaration, followed by its reaffirmation in 2018 by the Astana Declaration that recognized health as a human right.

Several issues have been discussed, including heightened competition for natural resources, poverty, reduced access to primary health care, ageing demographics, access to clean water and food and education, that need solutions that not only ensure the equitable distribution for the living population but also consider the needs of the future generations [1]. In the present healthcare policy perspective paper, we renew the uncountable calls for actions that have been made previously, especially from a healthcare viewpoint. We present an overview of the successfully implemented or drafted policy models from across the world that have been introduced in the recent past (albeit disregarding the negative impact of COVID-19) and what more needs to be done to achieve the sustainable developmental goals (SDGs).

## 1. Access to universal health coverage

**Figure 1.**
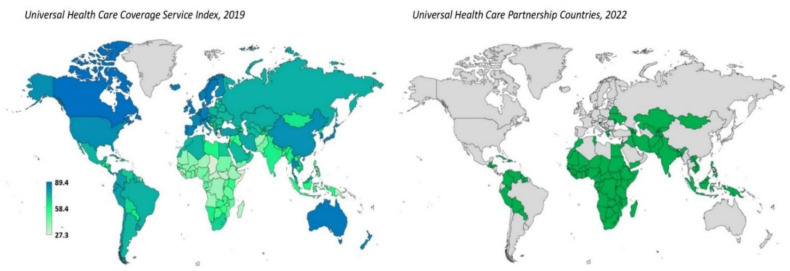
Global universal healthcare coverage service index for essential health services (0–100), 2019 (left). The higher the index, the higher the universal healthcare coverage (data source: https://www.who.int/data/gho/data/indicators/indicator-details/GHO/uhc-index-of-service-coverage; Accessed 26^th^ November 2022). Countries that are part of WHO's Universal Health Care Partnership (right) – a platform that works to deliver WHO's support and technical expertise in advancing universal health coverage (data source: https://extranet.who.int/uhcpartnership/countries; Accessed 26^th^ November 2022).

Universal health coverage (UHC) entails that everyone has access to the necessary healthcare without facing financial inconvenience, thereby offering a means of uniting national and international efforts to improve global health, combat poverty and reduce social inequality (Figure 1) [3]. It also serves as a proxy symbol of a government's dedication to enhancing the welfare of its residents. A growing population is often associated with the efficiency and advancement of the healthcare system.

However, rapid population expansion also puts a pressure on the public health infrastructure, reducing availability to essential care for individuals and degrading quality of life. The implementation of most current UHC programs have suffered significant setbacks stemming from lack of administrative coordination within the respective ministry of health´s overall structures, inadequate health information systems, lack of integrated clinical and frontline health worker data collection and synthesis networks, insufficient utilization and inaccurate interpretation of the available data, underfunding and poor quality medical facilities, thereby lowering the overall success of the programs. Experiences from sub-Saharan countries like Uganda have shown that health financing and risk prevention strategies protecting UHC reforms are far from the desired results [4].

It is equally pertinent to highlight the seldomly considered field of oral care, especially in the context of UHC. The recently published WHO's *Global oral health status report* (GOHSR; 18^th^ November 2022) aims to correct this ignorance by highlighting the burden, risk factors, health system challenges and opportunities for reform [5].

*“Oral health services are largely demand led, often poorly planned by public institutions, and influenced by entrepreneurial choices inherent to private practice...... Because independent private practitioners deliver a significant proportion of oral health care outside of the public health system, public health policymakers and politicians often consider dentistry to be a marginal issue of low priority.”*

*WHO GOHSR, November 2022*

To overcome these challenges, political and bureaucratic willpower needs to be established. There is a pressing need to develop self-sustained and rapidly adapting ecosystems that allow for interactive communication between government stewardship, stakeholder support and equitable resource contribution and distribution. Engagement of all concerned stakeholders including insurers and the person insured is a must. Public outreach and education play a crucial role as seen with the implementation of the US' Affordable Care Act and American Rescue Plan's that have led to significant increase in health insurance coverage during the 2021 and 2022, especially amongst the Black, Latinx, American Indian and Alaska Native communities [6].

Another interesting model is the Chinese model that strives towards guaranteeing health equity through increased technical advancements and enhancements to the health insurance. China launched one of the largest health system reforms almost a decade ago [7]. In the early 2000s, less than one out of three Chinese citizens had health insurance. Now, the coverage protects almost its entire 1.4 billion population. Essentially, China has been able to provide a safety net that guards against people becoming destitute due to the high expense of healthcare. By 2030, the Chinese government aims at creating a “healthy China” by a) prioritizing disease prevention and improving everyone's health; b) comprehensively reforming the medical and healthcare systems; c) generating of a green, safe and healthy environment; d) enhancing the health care sector and e) developing social policies and institutional systems for their proper implementation [7].

Neighboring India, set to be the most populous country by 2023, has also launched the Ayushman Bharat Pradhan Mantri Jan Arogya Yojana (PM-JAY) in 2018 that provides a cover of INR 500,000 (USD 6,100) for availing free secondary and tertiary healthcare services. The scheme is projected to provide cover to more than 500 million individuals [8]. Apart from the national initiatives, global initiatives like UHC2030 (https://www.uhc2030.org/) aim to strengthen health systems towards universal health coverage. It offers a venue for bringing people together and fostering relationships through collaborative high-level events or expert gatherings and contributes advocacy, resources, advice, expertise and learning. Other collaborative initiatives include the global biopharmaceutical giants-led Access Accelerated (https://accessaccelerated.org/), WHO-backed Providing for Health's (P4H's) Leadership for Universal Health Coverage (L4UHC; https://l4uhc.world/), and the Universal Health Coverage Coalition (http://healthforall.org/welcome/).

## 2. Shortage of healthcare providers

**Figure 2.**
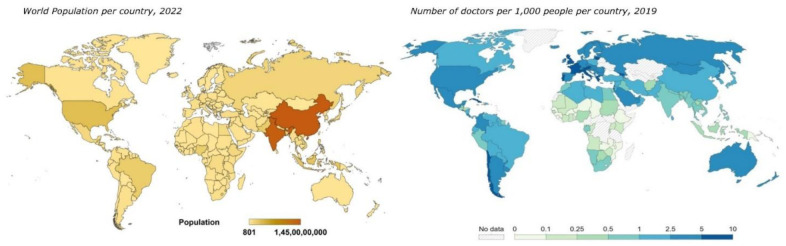
World population per country as of November 2022 (left; data source: Worldometer). Map depicting the number of doctors (generalists and specialists combined) per 1000 people per country as of 2019 (right; data source: https://ourworldindata.org/grapher/physicians-per-1000-people; Accessed 09^th^ December 2022).

Recognition and rectification of the global shortage of healthcare providers was one of the main agendas adopted in the political declaration of the high-level meeting on universal health coverage (UN General Assembly; September 2019), the first-ever such resolution to be passed [9]. According to WHO, the desirable doctor–population ratio is 1 doctor for 1,000 population. However, less than half of the countries satisfy this requirement (Figure 2) [10]. Not surprisingly, most avoidable and curable deaths also occur in these nations where healthcare is hard to access and people often live their whole lives without ever visiting a doctor or nurse [11].

Calls have been made time and again to support the need for governments and global health developmental partners to speed up investments in healthcare worker's education and facilitating the hiring of new healthcare providers. In 2020, there were an estimated 65.1 million healthcare providers worldwide, including 29.1 million nurses, 12.7 million physicians, 3.7 million pharmacists, 2.5 million dentists, 2.2 million midwives and 14.9 million other allied workers [12]. By 2030, the workforce is expected to reach 84 million worldwide, implying a 29% crude-rise over a 10-year period. While the global population growth rate is expected to be 9.7% over the same period, there would still be a future shortfall of 10–11 million healthcare professionals globally [12]. An often-advanced argument is to increase the number of medical schools and the capacity at existing schools. However, this move has proven to be counterproductive (evidenced by Canada, US) since the rise in undergraduate training positions does not correlate with the rise in residency and postgraduate training positions [13]. This, along with other factors like increased requirement of resources (technological, financial and expertise), has also led the UK government to reintroduce medical enrolment caps (7500 for 2022).

The persistent underinvestment in the education and training of healthcare providers in some nations as well as the misalignment of education and employment policies concerning health systems and population demands have led to a shortage of healthcare providers. COVID-19 has only exacerbated this shortage. Apart from the general shortage, challenges involved in recruiting medical personnel to underserved, rural and distant locations independently limit the reach of the healthcare system to masses. In a bid to alleviate the issue, HHS (Department of Health and Human Services, US) had set up the $750000 Rural Residency Planning and Development Program Grant in 2019 that would develop new rural residency programs for physicians. WHO-endorsed “grow your own” strategy has proven to be effective in retaining physicians in rural communities in countries like Australia and Japan that witnessed a higher rate of physician return to the same rural region as that of training by simply prolonging the training periods (>1 year) [14].

In certain countries, the public sector's inability to absorb the supply of health professionals because of financial restrictions may also contribute to problems with universal access to health workers. As a result, nations have a paradoxical situation of having significant unmet health demands and a shortage of health workers. The growing worldwide movement of health professionals, especially from lower and middle-income countries to high-income countries further adds to the misery. In 2014 alone, the top 20 countries with largest number of physician emigration (by crude count) collectively lost more than 350000 physicians from national workforces [15]. India, Pakistan, Germany, Philippines, Romania and nations in Caribbean, Central Asia and Eastern Europe have been the most affected in terms of physician emigration [15].

A significant threat to workforce shortage stems from gender inequalities [16]. It is estimated that only 25% of leadership positions in the global health sector are held by women, who make up approximately three-quarters of the healthcare workforce. In the US (as of 2020), only 15% of medical school deans were female, with most women being encouraged to take up nurturing roles like nurses, nurse midwives, community health workers and student affairs [17]. Such societal practices have tainted the reputation and credibility of nursing as a future career thereby contributing to a decrease in new yearly enrolments [17]–[18]. However, before implementing policies it is important to understand the scope of gender inequality and educate the concerned stakeholders. To this end, a pilot program “Medicine Goes Female” (MedGoFem) was initiated in 2018 in seven European countries to facilitate the implementation of gender equality plans (GEP) in local university hospitals [19]. In a bid to tackle the healthcare provider shortage, California has drafted a bill (Assembly Bill 890 (2022); pending voting) that will allow nurse practitioners to work unsupervised in the state if they meet the statutory requirements.

Finally, COVID-19 pandemic has accelerated burnout rates amongst healthcare providers. Burnout not only represents undergoing stressful, long, endurance-testing workhours but is the first step towards the fundamental disconnection from the motivation that propels providers to serve [20]. In fact, in 2020, about 20% of the surveyed US physicians and 40% of the US nurses intended to leave clinical practice altogether within the next two years due to the stressors of burnout [21]. This has led to the adoption of the 25x5 initiative in the US which aims to reduce the documentation burden on outpatient physicians to 25% of current loads within the next five years (by 2025). A similar initiative has been undertaken by Hawaii Pacific Health via their program “Getting Rid of Stupid Stuff” which has saved over 1700 nursing hours per month in the state's healthcare system [20]. To help caregivers cope with their psychological, mental and substance-use concerns, the landmark Dr. Lorna Breen Health Care Provider Protection Act was passed by the US Congress in March 2022.

## 3. Rising global burden of noncommunicable diseases (NCDs)

Noncommunicable diseases (NCDs) or chronic diseases are responsible for more than 70% (41 million) of global deaths yearly, leading to sharp rise in the associated morbidity [22]. Additionally, lack of treatment modalities and/or technology has led to a decline in quality of life and associated economic losses [23]. The majority of NCD deaths are associated with cardiovascular diseases, malignancy, chronic respiratory diseases and diabetes. Other widely prevalent NCDs include psychological and reproductive disorders.

Current and future strategies on NCDs prevention should focus on a multiprong strategy. Ideally, strategy should take advantage of opportunities at all life stages by limiting risk factors, managing NCDs, preventing progression of NCDs and protecting for recurrence or multimorbidity. Research is key to future progress on NCDs management. NCDs should be seen as a long-term problem that cannot be solved through a single, time-limited commitment but rather calls for long-term interventions and sustainable solutions.

Since the majority of NCDs fatalities worldwide occur in low and middle-income countries (LMICs), it has only expanded the economic and health inequalities with developed countries [24]. One of the reasons could be that people living in high-income countries (HICs) have better access to health care for timely diagnosis and treatment, both in terms of finances and transportation [25]. On the other hand, LMICs face two major problems. First, while targets should be thoroughly monitored and evaluated, research infrastructures and outputs in LMICs are inadequate and there is hence a lack of data from LMICs [26]. Second, LMICs lack the financial means to cover the costs of preventive care.

Solutions lies in ways to promote the efficiency of NCD services delivery. While current healthcare practices are mainly focused on acute care and infectious diseases, healthcare settings should really be prepared for prevention and increased chronic care and focus on enhancing patient compliance and self-management [27]. Implementation of prevention measures for NCDs can be done by prioritizing NCDs interventions based on equity, safety and cost-effectiveness and healthcare providers must follow prescription and investigations requests based on evidence-based and cost-effectiveness [26]. This necessitates the creation of local research infrastructure and skilled professionals, both requiring increased financial resources. Prevention and controlled management of NCDs will likely lead to decreased healthcare costs [28]. This has also been illustrated by the recent analyses across 23 LMICs which showed that institutional delivery and participation in antenatal care programs are strongly and positively correlated with high-quality post-natal care, thereby reducing maternal and neonatal mortality [29].

**Figure 3.**
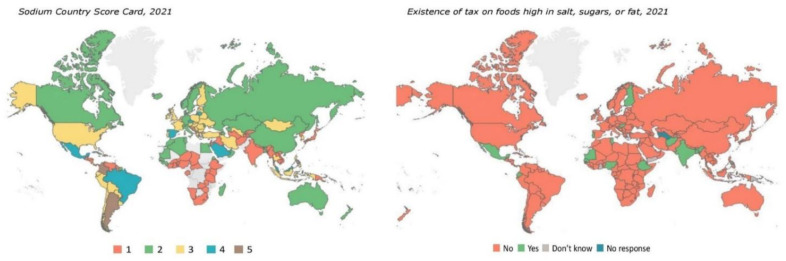
WHO's Sodium Country Score Card to track countries' performance in implementing various measures for reducing salt intake (left). 1. National policy commitment to reduce sodium intake; 2. Voluntary measures to reduce sodium; 3. Mandatory measures adopted for sodium reduction; 4. Multiple mandatory measures adopted for sodium reduction, and implementation of all related WHO Best Buys for tackling NCDs; 5. Mandatory measures for sodium reduction adopted but not yet implemented (data source: https://extranet.who.int/nutrition/gina/en/scorecard/sodium). Global map depicting whether the country has implemented taxation on foods high in fat, sugars or salt (right; data source: https://www.who.int/data/gho/data/indicators/indicator-details/GHO/existence-of-tax-on-foods-high-in-fat--sugars-or-salt).

While a lot of cost-effective prevention methods for NCDs are well described, the real challenge is in their proper implementation [30]. For example, 1.8 million deaths are registered annually due to NCDs caused by excess salt-intake that prompted WHO in 2013 to advise all member states to cut population's salt consumption by 30% by 2025. While countries have been increasingly implementing practices to lower salt intake including reformulation of food, education of consumers, front-of-pack labelling schemes, salt taxation and interventions in work and school settings (Figure 3) [31], no country has reached the target. On December 9^th^, 2022, a new WHO/Europe signature initiative to tackle cardiovascular disease (CVD) was launched that will promotes education and implementation of various preventive practices [32]. It was one of the six signature initiatives associated with the Regional Director's Advisory Council on Innovation for NCDs.

Leading by example in resource-constricted settings, the Pan American Health Organization (PAHO) has been working to improve physical, mental and social well-being through lowering risk and the disease burden by enhancing public awareness and understanding of most prevalent NCDs and associated risk factors, lead multi-stakeholder collaborations and promote health by programs, services and public policies. Interventions as simple as reading books to and with children, telling them stories and counting or drawing with them are associated strongly with on-track literacy-numeracy development in the early childhood phase [33].

Several studies have shown that lifestyle interventions in primary care, including physical activity on prescription (PAP), can improve quality of life and are more cost-effective, compared to standard care [34]–[35]. An example of such lifestyle intervention program comes from Sweden. The Swedish model of PAP enables health care professionals to prescribe exercises based on intensity, duration, and type of activity the patient should perform to optimize their health. The success of the program has led to its coordinated introduction in nine other European countries-Belgium, Denmark, Germany, Italy, Lithuania, Malta, Portugal, Romania and Spain (EUPAP; https://www.eupap.org/).

## 4. Ageing population and changing demographics

In the past three decades, the worldwide proportion of individuals aged 65 and higher has risen from 6.1% in 1990 to approximately 10% in 2022, a trend that is projected to continue. By 2050, the geriatric proportion is expected to be around 16% or 22% based on WHO's estimates [36]–[37]. Population aging has numerous consequences, primarily from a socioeconomic and health standpoint. One of the most significant burdens is an increase in healthcare costs in chronic disease patients, particularly when they lack savings, insurance or pension funds [38]. Chronic disease patients require continuous funding to cover their medication, laboratory tests, healthcare consultations, use of long-term care facilities and medical devices such as hemodialysis [39].

Because of the smaller percentage of working and productive individuals living in society, a shift in demographic structure toward an older population is linked to a rise in the dependency ratio [40]. Furthermore, this causes a reduction in the contributions to the state social security, thereby limiting the spending power of the governments. Hence the governments and other representatives should take the opportunity to engage in health promotion and disease prevention programs, which are regarded as less expensive alternatives due to their position as primary prevention, rather than continuing to work in secondary or tertiary level preventative measures. Physical activity endorsement, stress and social management, alcohol and smoking cessation and healthy eating are all prevention objectives.

Instead of the current fragmented approach in which disease-based task problems are employed for noncommunicable diseases, cancer and mental health issues in the elderly, the intervention in health care for the elderly necessitates a holistic strategy [41]. Vaccination against preventable infections for risk groups like those with autoimmune conditions is a good example [42]–[43]. Furthermore, transitioning from conventional fee-for-service healthcare to packaged, capitated and other utility payments is highly demanded [44]. This model has already been implemented in some developing countries, such as Indonesia, with the establishment of the Social Security Agency of Health (*Badan Penyelenggara Jaminan Sosial Kesehatan*/BPJS), which employs all elements of prevention, early detection, and screening programs, as well as treatment, as part of the chronic disease management program (*Program penanggulangan penyakit kronis*/Prolanis). BPJS positively impacted the community's health and economic variables, primarily due to an increase in health awareness, lower healthcare costs and decreased irrational self-medication [45].

In the meantime, innovative healthcare must also be supported, particularly by incorporating technology, such as telemedicine and video-supported medicine [46]. India has recently rolled out a nationwide tele-mental health program called Tele Mental Health Assistance and Networking Across States (Tele-MANAS) which provides free, round-the-clock teleconsultation service for mental health concerns. Similarly, based on their previous successes, the European Commission (EC) and WHO Europe have approved several initiatives including the Regional Digital Health Action Plan for 2023–2030 to develop and implement telemedicine [47].

Additionally, health education is essential for both primary prevention (restricting disease emergence) and secondary/tertiary prevention (especially in the form of routine control to physicians and maintenance of medication adherence to reduce complication risk) [48]–[49]. Another critical issue is ensuring universal health coverage (UHC) for all seniors around the globe. The main intention is to avoid catastrophic expenses (due to hospital stays and doctor appointments) and to grant healthcare access, particularly to the sick [50]. However, it must be accompanied by a community awareness to seek medical attention as well as more substantial customization of perks to satisfy the health needs of this demographic [51].

Regarding the convergence of population aging and growing prevalence of noncommunicable diseases necessitating protracted and expensive care, a shift toward a more regenerative medicine strategy is critical [52]. Regenerative medicine has the potential to profit people because of its fundamentals of assisting in the rejuvenation of impaired body functions, providing the necessary elements for in vivo restoration, designing substitutions that flawlessly engage with the body system and stimulating the body's intrinsic capabilities for healing [53], which cannot be accomplished through conventional drug usage. However, the present situation still poses a significant challenge to the execution of regenerative medicine and cell therapy due to a lack of government support, definitive regulation, ethical and objectionable issue of cell technologies and insufficient funding, particularly in developing countries [54]–[55]. Consequently, broad adoption is becoming restricted regarding disease targets or administration techniques.

Another major demographic trend seen is the rapid rise in the proportion of the population residing in the urban areas. Rapid urbanization without concurrent planning and development of required infrastructure have led to difficulties in providing sufficient housing, healthcare and educational facilities to the resident population, alongside promoting violence and crime. Pollution, unsanitary living conditions and overcrowding exert detrimental effects on the health of the population. Urban migration is expected to continue due to limited resources in the rural setting, particularly in the developing countries.

At the same time, rapid urbanization can lead to improper vector control measures, particularly in urban slums. Poor living conditions in urban slums has also been attributed to an increased risk of vector-borne disease such as malaria and dengue [56]–[57]. Lymphatic filariasis, usually confined to rural areas, is also being reported in urban settings due to improper housing conditions caused by the population explosion [58]. Other such vectors/diseases include West Nile virus, Chagas disease and leishmaniasis.

Proper planning of housing space, infrastructure and promotion of social protection will serve to mitigate the worsening quality of life in urban areas. Overpopulation has also placed a strain on employment opportunities in many countries, thereby drastically increasing migration to other countries. According to the World Health Organization, more than 3% of the world population is residing in countries other than their native country. Migrant workers often endure unsanitary living conditions and hazardous work environments to earn minimal wages. Migrants are also at excessive risk of being exploited by human traffickers. International collaboration is, hence, crucial in ameliorating the health problems faced by migrant workers.

**Figure 4.**
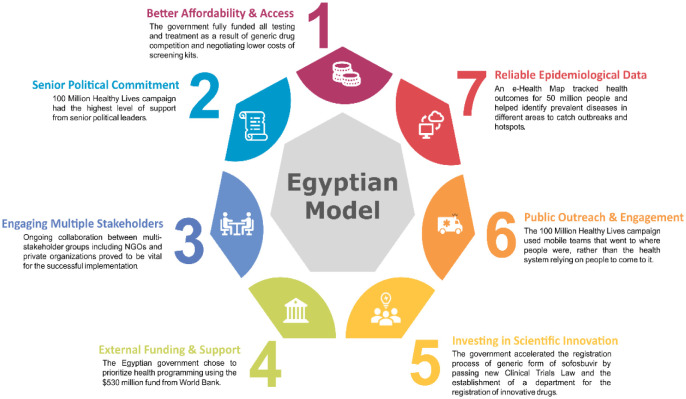
Factors that led to the success of the Egyptian model of combating Hepatitis C infection in the country “100 million Healthy Lives Campaign”. The factors were analyzed and compiled in a white paper summary by the World Economic Forum (WEF).

Hepatitis is a common infectious condition seen in migrant workers. Though the high burden is representative of the high disease burden in the native countries of migrants, migration does promote vulnerability of migrant workers towards being targeted by drug dealers [59]. This also makes them prone to longer and frequent police arrests, thereby increasing the risk of viral Hepatitis C (HCV) infections. The Egyptian model of combating Hepatitis C (100 million Healthy Lives campaign) has been hailed as a landmark feat (Figure 4). Egypt has been able to screen more than 60 million people and treated 4 million people over the past eight years, putting it on track for being the first country to eliminate HCV within its borders [60].

## 5. Access to portable water and food

A growing population creates numerous problems with respect to water and food shortages as well as water contamination both directly and indirectly. Given that currently 1.6 million deaths per year are attributed to consumption of unsafe water, access to portable water is already a significant problem [61]. Estimates suggest that greater than 50% of the world's population will have limited supplies of clean water for more than or equal to one month by 2050 [62]. Rapid urbanization, sandmining, agriculture, inadequate disposal of waste materials as well as climate change have and will only accelerate this shortage.

It has been projected that half of the global population will experience issues with water quality within the next 10 years, with more and more people being forced to drink sewage-contaminated water [61]. This poses a significant threat to human health by increasing the likelihood of developing water-borne diseases such as cholera. Waterborne diseases contribute a significant load to healthcare system in both developed and developing countries [63]. Lack of access to safe drinking water can lead to increased occurrence of soil-transmitted helminthic (STH) infections like *Ascaris lumbricoides* and *Trichuris trichiura* [64]–[65]. The situation is further precipitated due to increased contamination with industrial chemicals, solvents and pesticides.

Water shortages considering a growing population will also negatively impact on food availability. It has been projected that by 2050 the effects of a growing population and climate change will result in an increase of 30% of the global population being at risk of hunger [66]. As a result, it is predicted that 120 million people will suffer from malnourishment by 2080. An increased prevalence of transboundary diseases because of a growing population and migration will also lead to a reduction in food productivity, given that presently transboundary diseases result in an approximate 40% loss in global food production [67].

There is clearly a need for encouraging a more sustainable future through strategies that increase cropping efficiency and improve land cultivation including using high-throughput single nucleotide polymorphism (SNP) genotyping. International technology transfer and funding is essential for capacity building in developing countries. For example, the Water Scarcity Regional Initiative (WSI) from UN aims to addresses issues of food and water insecurity in the Middle East and North Africa (MENA) region sustainably, using techniques like integration of agriculture-aquaculture pilot-farms in Africa. Regarding water contamination and food shortages, Water Aid's ‘Everyone, Everywhere 2030’ Strategy encourages governments to ensure that everyone gets access to clean water by 2030.

## 6. Our world, our future

Although there are many disadvantages that a growing population will bring, the inevitable growth of the global population also provides an opportunity to the governments to implement more sustainable strategies to address the needs of today and tomorrow. Hence, it is crucial to adopt strategies that blend inclusive participation of communities while preserving their cultural and historic knowledge.

According to WHO, around 88% of all countries are estimated to use traditional medicine and/or other forms of complementary medicine (T&CM) [68]. In the light of the shortage of western medicine practitioners (as discussed above), allying with traditional medicine care providers not only boosts the reach of the healthcare system, but also ensure compliance, trust and helps to monitor disease outbreaks [65]. In this regard to promote and safeguard use of safe practices in traditional medicine, WHO set up its first Global Centre for Traditional Medicine (GCTM) in 2022 in India [69]. Additionally, many countries have enacted laws integrating T&CM into their national health services to boost the outreach of universal health cover. Examples include Malaysia's Traditional and Complementary Medicine (T&CM) Act of 2016, India's AYUSH (ayurveda, yoga and naturopathy, unani, siddha and homeopathy) system and Uganda's Traditional and Complementary Medicines Act of 2019.

Clearly, the implementation of sustainable strategies and ensuring inclusivity requires innovative and out-of-box solutions. Leading by example, Health in Harmony (HIH), a nonprofit organization has blended rainforest conservation with healthcare promotion amongst native communities in Indonesia, Brazil and Madagascar. Their use of innovative “radical listening” technique uses a form of barter-exchange-establishing medical center with discounts to communities along with alternative payment methods such as seedlings, trainings, etc., in return for protecting rainforests by reforestation and reducing illegal lodgings [70].

Despite these efforts, wealth continues to play a key but disproportionate determinant which influences the viability of these strategies. Whilst developing countries suffer from the ill-consequences of overpopulation and struggle to introduce meaningful reforms, the developed countries continue to underserve on their commitments on climate action, capacity building, technology sharing and funding pledges.

*“We now find three gaps–* (on finance)*, mitigation, climate pledges or NDCs* (Nationally Determined Contributions) *...... How many more voices and how many more pictures of people must we see on these screens without being able to move?....... Can there be peace and prosperity if one third of the world literally prospers and the other two thirds of the world live under siege and face calamitous threats to our well-being?”*

*- Barbados Prime Minister H.E. Mia Amor Mottley, COP26, 2019*

## Use of AI tools declaration

The authors declare they have not used Artificial Intelligence (AI) tools in the creation of this article.
